# CMPF: Class-switching minimized pathfinding in metabolic networks

**DOI:** 10.1186/1471-2105-13-S17-S17

**Published:** 2012-12-07

**Authors:** Kevin Lim, Limsoon Wong

**Affiliations:** 1 School of Computing, National University of Singapore, Singapore

## Abstract

**Background:**

The metabolic network is an aggregation of enzyme catalyzed reactions that converts one compound to another. Paths in a metabolic network are a sequence of enzymes that describe how a chemical compound of interest can be produced in a biological system. As the number of such paths is quite large, many methods have been developed to score paths so that the k-shortest paths represent the set of paths that are biologically meaningful or efficient. However, these approaches do not consider whether the sequence of enzymes can be manufactured in the same pathway/species/localization. As a result, a predicted sequence might consist of groups of enzymes that operate in distinct pathway/species/localization and may not truly reflect the events occurring within cell.

**Results:**

We propose a path weighting method CMPF (Class-switching Minimized Pathfinder) to search for routes in a metabolic network which minimizes pathway switching. In biological terms, a pathway is a series of chemical reactions which define a specific function (e.g. glycolysis). We conjecture that routes that cross many pathways are inefficient since different pathways define different metabolic functions. In addition, native routes are also well characterized within pathways, suggesting that reasonable paths should not involve too many pathway switches. Our method can be generalized when reactions participate in a class set (e.g., pathways, species or cellular localization) so that the paths predicted have minimal class crossings.

**Conclusions:**

We show that our method generates k-paths that involve the least number of class switching. In addition, we also show that native paths are recoverable and alternative paths deviates less from native paths compared to other methods. This suggests that paths ranked by our method could be a way to predict paths that are likely to occur in biological systems.

## Background

Metabolic networks consist of small chemical molecules that are transformed from one to another by enzymes in a specified series of reactions. Predicting source-to-target routes in metabolic pathways is an interesting problem that has applications in synthetic biology, bioengineering and systems biology. For example, a biologist might be interested to know how long-chained lipids might be produced. It is also useful in tracer and genetic knockout experiments [1].

The problem is defined on a set of rules which dictate how substrates are transformed into products on a given enzyme. Blum and Kohlbacher described two early approaches targeted at this problem [1]. The first models compound transforming rules into a graph and employs shortest path algorithms to predict routes [2,3]. The second approach expands a set of compounds (initially just the starting substrate) by applying the set of rules iteratively adding the products of reactions to the set [4]. A shortest path algorithm is then run on a graph constructed from the resulting set of compounds.

It is uninteresting to only output a single shortest path, because native paths seldom have the shortest topological length. For example, the path from glucose to pyruvate has a shortest length of 4, but the native path requires 9 reactions. On the other hand, the number of paths between a source and a target can grow exponentially in a highly connected network. For example, Blum and Kohlbacher reported that between glucose and pyruvate, there can be about 500,000 paths [5]. Most reported methods use Eppstein's k-shortest path algorithm [6] in which the paths are not guaranteed to be simple loopless paths. Routes with loops are biologically uninteresting because they overlap with a shorter route previously discovered. The problem of finding k-shortest simple loopless paths incurs higher complexity and is harder to implement. The results from shortest-path finding is also highly dependent on the weights associated with nodes or edges, which are modelled differently in different approaches. For example, Croes et al. use nodes to represent chemical compounds and assigned weights to nodes based on its degree centrality [2]. Rahman et al. assign weights to edges based on compound structure similarity [3].

Earlier approaches also try to avoid compounds participating in many reactions but do not play an important role in the path. These compounds are termed as 'currency' compounds since they are circulating in metabolic pathways. Blum and Kohlbacher use atom mapping rules which keeps track of atom transfer between substrate and product compounds to avoid paths with currency compounds [1]. More recent approaches relied on new information -- e.g., the RPAIR database [7-10] -- to avoid 'currency' compounds and to infer more reliable routes. Xia et al. use species information to model the weights of graph edges [11], they believe that reactions that can be found in more species are more reliable. However, these approaches do not check whether it is possible to start with a reaction from a certain pathway/species and end with a reaction so that as little change in pathway/species is required in the route. It might be biologically unmeaningful to obtain routes where there are reactions in the middle of the route that cannot be produced from the pathway/species that the first substrate started from. We think that routes with minimal switches are more preferable. In addition, most approaches fail to recover the native source-to-target route that is used by biological systems. We suggest that plausible routes should not deviate too much from native routes.

**Figure 1:**
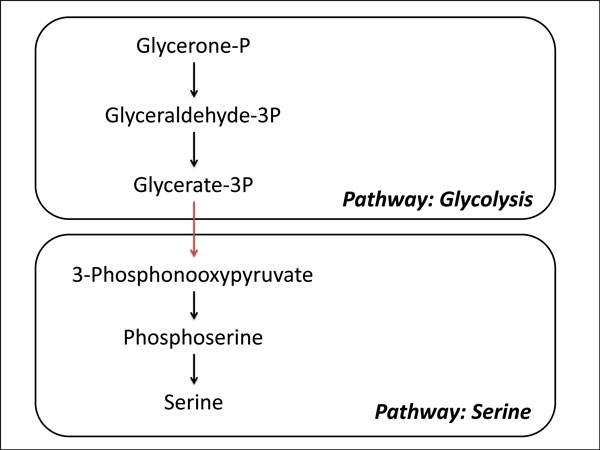
**A pathway-switching route crossing the glycolysis/serine pathway**. A pathway switch occurs when an enzyme catalyzed reaction exist in a separate pathway from precursor reactions. In this example, the enzyme that catalyzes the reaction from Glycerate-3P to 3-Phosphonoexypyruvate does not exist in the glycolysis pathway.

In this paper, we propose a method, Class-switching Minimized Path Finder (CMPF), to find *k *routes that minimizes species/pathway switching. A switch is the case when a reaction in a path leads from a plausible set of species/pathways to a distinct set of species/pathways (See Figure 1). The routes are scored so that routes that cross many species/pathways have a higher penalty than those that do not.

## Methods

### Basic Framework

One way to find paths that minimizes species crossing is to reduce this problem to the original k shortest path problem. This can be done by modelling the network using a reaction connecting graph where by the nodes are reactions and an edge connects two reactions if the product of one is the substrate of the other. Unique reaction nodes are duplicated for each species that the reaction occurs in. For example, if ten species are involved in a reaction, then ten separate reaction nodes will be created. An edge is assigned weight of zero if the two nodes have the same supporting species and one otherwise. Any k-shortest path produced by the algorithm also has minimum species crossing because each cross-species reaction in the path has a penalty of one, whereas reactions that do not cross species have no penalty. However, this results in a prohibitively large number of nodes and edges. For example, if every reaction is supported by 1000 species on average, then for every two reaction nodes in the original graph, we obtain 2000 nodes with 1,000,000 edges between them.

**Figure 2:**
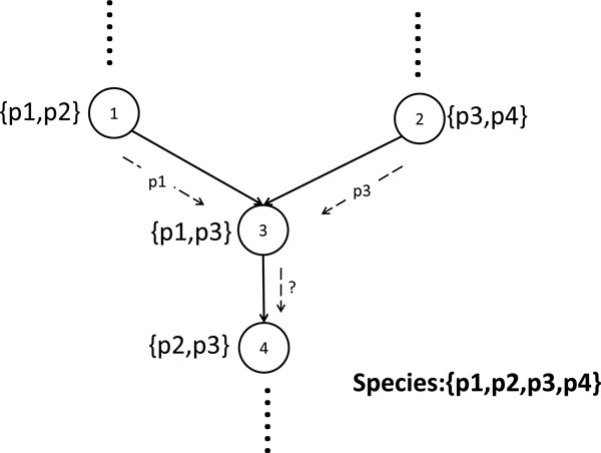
**An example demonstrating that k-shortest path cannot be used to find paths with minimal-switching**. The nodes represent reactions and are annotated with species in which the reaction exist. The path on the left requires species p1 and the path on the right requires speices p3. Reaction nodes 3 and 4 are shared between the two paths. The path on the left incurrs a switch and the path on the right incurrs no switch. The switching requirement cannot be captured using edge weights assigned to the edge 3,4. Instead, the score of each paths should be computed separately.

If the reaction nodes are compacted by storing all the species supporting that reaction, then a k-shortest-path approach does not work. This is because two routes might share a common reaction and a switch is incurred in one of the routes but not the other. On the other hand, a k-shortest-path approach requires the edge weight to be unchanged. When the shared reaction has a shared edge weight, a switch cannot be captured by such a framework. This suggests that paths should be scored independently from one another (See Figure 2).

Instead, the algorithm in CMPF uses bounded depth path enumeration and scores the paths based on a scoring scheme where paths that cross species/pathways many times have higher penalty scores than those that do not. We use a bipartite graph to model the metabolic network which consists of RPAIR nodes *r*_1_, ..., *r_n _*and compound nodes *c*_1_, ..., *c_n_*. An RPAIR is pair of compounds with similar chemical structure on two sides of a reaction [7]. A directed edge connects RPAIR to compound and vice versa if the compound participates in the reaction represented by the RPAIR. We use the notation RPAIR and reaction interchangeably in this paper, since they represent similar concepts.

**Figure 3:**
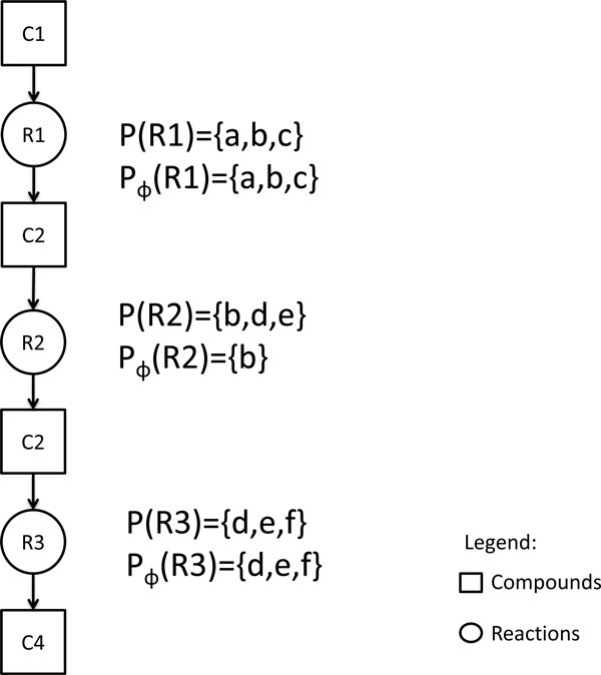
**An example illustrating the sets *P_ϕ_*(*r*) and *P*(*r*)**. A bipartite graph showing reactions r1, r2, r3 and compounds c1, c2, c3. The transition from r1 to r2 can be made by using pathway b but the transition from r2 to r3 requires a switch from pathway b to one of the pathways d,e,f.

Given a reaction *r_i _*in a linear path *ϕ *= *r*_1 _→ ... → *r_n_*, we write *P_ϕ _*(*r_i_*) and *S_ϕ _*(*r_i_*) respectively to be the set of pathways and species that "support" the reaction *r_i _*in the linear path *ϕ*. We assume that the pathways and species that support the first reaction *r*_1 _of *ϕ *are all the pathways and species that *r*_1 _belongs to; that is, *P_ϕ _*(*r*_1_) = *P*(*r*_1_) and *S_ϕ _*(*r*_1_) = *S*(*r*_1_). For an intermediate reaction *r_i _*in the linear path *ϕ*, if possible, the pathways and species that support it should be those that *r_i _*belongs to and also support the preceding reaction *r*_*i *- 1 _. Thus, *P_ϕ _*(*r_i_*) = *P_ϕ _*(*r*_*i *-1_) ∩ *P*(*r_i_*), provided *P_ϕ _*(*r*_*i*-1_) ∩ *P*(*r_i_*) is nonempty; and *S_ϕ _*(*r_i_*) = *S_ϕ _*(*r*_*i *- 1 _) ∩ *S*(*r_i_*), provided *S_ϕ _*(*r*_*i *- 1 _) ∩ *S*(*r_i_*) is nonempty. On the other hand, when the set of pathways or species that support the preceding reaction *r*_*i *-1 _is totally different from the set of pathways or species that *r_i _*belongs to, it is not possible to transition from reaction *r*_*i *-1 _to *r_i _*in the linear path *ϕ*. That is, in this case, a pathway or species switch is necessary. We assume that the entire set of pathways or species that *r_i _*belongs to can be used for this switch. Thus, *P_ϕ _*(*r_i_*) = *P*(*r_i_*) when *P_ϕ _*(*r*_*i *-1_) ∩ *P*(*r_i_*) is empty; and *S_ϕ_*(*r_i_*) = *S*(*r_i_*) when *S_ϕ _*(*r*_*i *-1_) ∩ *S*(*r_i_*) is empty (See Figure 3).

Now, we are ready to define our basic framework of reaction transition weight and linear path score as follows:

**Definition 1 ***The weight of a transition r_i _*→ *r*_*i*+1 _*in a linear path ϕ *= *r*_1 _→ ... → *r_n _based on pathways is defined as*

$$weight_{P,\phi }\left(r_{i}\to r_{i+1}\right)=\gamma +\delta ,$$

*where γ is some constant denoting the cost of making a within-pathway transition and δ is the extra cost of making a pathway switch; note that δ *= 0 *if P_ϕ_*(*r*_*i *-1_) ∩ *P*(*r_i_*) *is nonempty, as a pathway switch is not needed in this situation. We will further refine γ and δ in the next section*.

The weight of a transition *r_i _*→ *r*_*i*+1 _based on species is denoted *weight_S,ϕ _*(*r_i _*→ *r*_*i *+1_), which is defined analogously.

**Definition 2 ***The pathway- or species-based score of linear path ϕ *= *r*_1 _→ ... → *r_n _is just the sum of the weights of all the transitions in ϕ based on pathways or species. That is*,

$$score\left(\phi \right)=\overset{n-1}{\underset{i=1}{\sum }}weight_{\phi }\left(r_{i}\to r_{i+1}\right)$$

*where weight_ϕ _*(*r_i _*→ *r*_*i*+1_) *is either weight *_*P*,*ϕ *_(*r_i _*→ *r*_*i *+1_) *or weight *_*S*,*ϕ *_(*r_i _*→ *r*_*i*+1_), *for pathway*- *or species-based score respectively*.

In our scoring scheme, the weight of a transition *r_i _*→ *r*_*i*+1 _in a linear path depends on whether the pathways (or species) that support *r_i _*also support *r*_*i*+1 _in that specific linear path. Thus, a topologically shorter linear path may have a higher score (i.e., cost) than a longer one. Moreover, the computation of the score is independent between different linear paths, suggesting exhaustive enumeration of linear paths as a method to find and rank linear paths. Since most linear paths that are useful usually do not exceed a certain topological length and also because exhaustive enumeration can be slow, we use bounded depth enumeration to speed up the search process.

We construct a global metabolic bipartite graph annotated with species/pathway information and exclude non-main RPAIRs to avoid 'currency' compounds. We enumerate all possible paths up to a specified depth and return the *k *lowest scoring linear paths based on our scoring scheme. In this way, we guarantee that linear paths generated by our method have the least number of cross-pathway/species reactions and are always optimal up to the depth to which we traverse the graph.

### Framework Extensions

#### Edge Weights

Many other approaches have tried giving paths meaning by defining meaningful edge weights. Our framework allows us to reuse these ideas and incorporate these weights. For example, Croes et al. use node connectivity as edge weights so that 'currency' compounds are avoided [2]; and Xia et al. use the inverse of organism frequency that a reaction belongs to as edge weights so that reactions that belong to more organisms are preferred [11]. In CMPF, we use *γ*_1 _and *γ*_2 _to denote these two scoring strategies.

#### Penalty Scores

Native paths rarely involve a switch between pathways. On the other hand, alternative paths might involve such switching. The penalty given when a switch occurs can be made more meaningful, since some pathway crossing is preferable to others. Our framework discussed thus far allows us to rank linear paths by their pathway switching equivalence class. A pathway switching equivalence class here refers to a group of linear paths with the same number of pathway/switches. However, the arbitration of linear paths within the same pathway switching equivalence class is random.

We can do better by defining a function which computes the distance from one pathway to another. We define the 'metabolite closure' M(x) of a pathway x to be the set of metabolites that are generated within that pathway. We hypothesize that pathways performing similar function have similar metabolite closures because the end product often determines intermediate metabolites. Hence, a switch from pathway x to pathway y would be preferred if their metabolite closures agree well with each other.

**Definition 3 ***The distance between two pathways x and y is the average of the normalized size of the set difference of their metabolite closures M(x) and M(y)*.

$$\delta_{1}^{\prime }\left(x,y\right)=0.5*\frac{M\left(x\right)\backslash M\left(y\right)}{M\left(x\right)}+0.5*\frac{M\left(y\right)\backslash M\left(x\right)}{M\left(y\right)}$$

In addition, a switch can involve two sets of pathways. In this case, we take the average distance of all possible switchings.

**Definition 4 ***Given a transition r_i _*→ *r*_*i *+1 _*in a linear path ϕ and P_ϕ_*(*r_i_*) **∩ ***P*(*r*_*i*+1_) *is an emptyset, we define*

$$\delta_{1}=\frac{\sum_{x\in P_{\phi }\left(r_{i}\right),y\in P\left(r_{i+1}\right)}\delta_{1}^{\prime }\left(x,y\right)}{\left|P_{\phi }\left(r_{i}\right)\times P\left(r_{i+1}\right)\right|}$$

*and δ*_1 _= 0 *if P_ϕ _*(*r_i_*) ∩ *P*(*r*_*i*+1_) *is non-empty*.

Similarly, a switch between species is more likely to happen if the two species are related. The distance between two species can be similarly defined using their metabolite closures. A more intuitive measure of species similarity is one that is based on the taxonomy tree. In this case, the path length from species x to y in the taxonomy tree can be used to determine the distance between them; we denote this distance by $\delta_{2}^{\prime }\left(x,y\right)$.

**Definition 5 ***Given a transition r_i _*→ *r*_*i*+1 _*in a linear path ϕ and S_ϕ _*(*r_i_*) ∩ *S*(*r*_*i*+1_) *is an emptyset, we define*

$$\delta_{2}=\frac{\sum_{x\in S_{\phi }\left(r_{i}\right),y\in S\left(r_{i+1}\right)}\delta_{2}^{\prime }\left(x,y\right)}{\left|S_{\phi }\left(r_{i}\right)\times S\left(r_{i+1}\right)\right|},$$

*which is the average taxonomic path length between the two sets of species that support these two consecutive reactions in the linear path ϕ*; *δ*_2 _= 0 *if S_ϕ _(r_i_) *∩ *S(r*_*i*+1_) *is non-empty*.

#### Combination of Scoring Functions

We modify Definition 1 to a weighted sum of scores defined in the previous section.

**Definition 6 ***The weight of a transition r_i _*→ *r*_*i *+ 1 _*in a linear path ϕ = r*_1 _→ ... → *r*_*n *_*is defined as*

$$weight_{\phi }\left(r_{i}\to r_{i+1}\right)=w_{1}*\gamma_{1}+w_{2}*\gamma_{2}+w_{3}*\delta_{1}+w_{4}*\delta_{2}$$

*where γ*_1_, *γ*_2_, *δ*_1_, *and δ*_2 _*are described earlier*; *and w*_1 _+ *w*_2 _+ *w*_3 _+ *w*_4 _= 1.

## Results

### Constant Penalty

We compare CMPF using standard reference metabolic pathways as defined in [12] against MRSD [11], MetaRoute [1] and a pathfinding method in the NEAT software package described in [2]. MRSD and MetaRoute use compound transform graph to model the metabolic network and use species support and node connectivity respectively to score paths. NEAT model the metabolic network using RPAIRs and the score of a path is a combination of the node connectivity and a score given for each type of RPAIR. Our framework can easily model these approaches. For example, we can easily emulate MRSD by setting *w*_1 _= *w*_3 _= *w*_4 _= 0 in Definition 3. It can also easily emulate MetaRoute by setting *w*_2 _= *w*_3 _= *w*_4 _= 0. At the same time, we can avoid pathway/species switches while emulating these previous approaches. We define *weight_ϕ_*(*r_i _*→ *r*_*i*+1_) as *w*_1 _* *γ*_1 _+ *w*_2 _* *γ*_2 _+ *δ*. We use *γ*_1 _(node connectivity) and *γ*_2 _(species support) defined in section 3.1 and set *δ *to be 100, to avoid penalizing long routes with no switching. We set *w*_1 _and *w*_2 _to be 0.5 so that *γ*_1 _and *γ*_2 _have equal contribution to the edge weight. For example, a route without switching will only be ranked lower than a switching path if its topological length is greater than 100. The top 20 paths for each method is obtained and the number of pathway switches in every path measured. For each reference case, we computed the average number of switches; see table 1. We observe that most paths returned by other methods incur many pathway switches.

**Table 1:** Comparing average number of pathways crossed from the top 20 routes in two methods.

Source	Target	switch*_mrsd_*	switch*_neato_*	switch*_metaroute_*	switch_*cmpf*1_	switch_*cmpf*2_
glucose	pyruvate	1.95	1.5	2.95	0	0.85
glutamate	proline	2.45	2.79	0.47	0	0.3
oxaloacetate	malate	1.75	1.33	0.65	0	0.95
glutamate	arginine	2.45	2.05	0.4	0	0
cdp-diacyl-glycerol	cardiolipin	1.75	1.0	1.8	0	0.1
gtp	riboflavin	2	1.78	4.25	0.95	1.05
allantoin	glyoxylate	4.3	2.0	*	1.85	3.15
rhamnose	pyruvate	1.9	0.875	1.1	0.85	1

*CMPF*_1 _uses static penalty and *CMPF*_2 _uses dynamic penalty. (*denotes no path found by method)

### Dynamic Penalty

**Figure 4:**
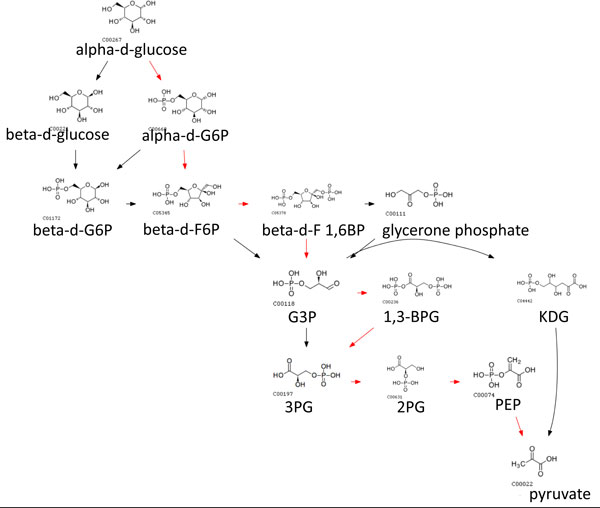
**Top 10 routes from glucose to pyruvate obtained using CMPF with dynamic penalties**. Paths predicted using CMPF with dynamic penalties deviate the least from the native path highlighted in red compared to other methods. As compared to using constant penalties, using dynamic penalties may permit a switch if the species/pathways involved in the switch are similar to each other. Dynamic penalties can also differentiate two paths with equal number of switches.

**Figure 5:**
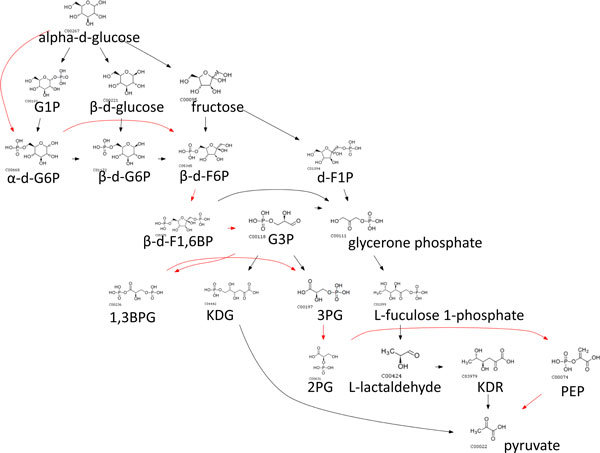
**Top 10 routes from glucose to pyruvate obtained using CMPF with constant penalties**. Paths predicted using CMPF with static penalty where the cost of a switch is set to 100. The native path is highlighted in red.

**Figure 6:**
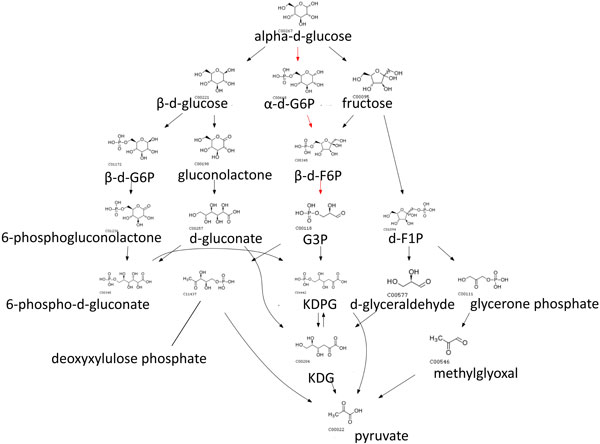
**Top 10 routes from glucose to pyruvate obtained using MRSD**. (Native path not recovered).

**Figure 7:**
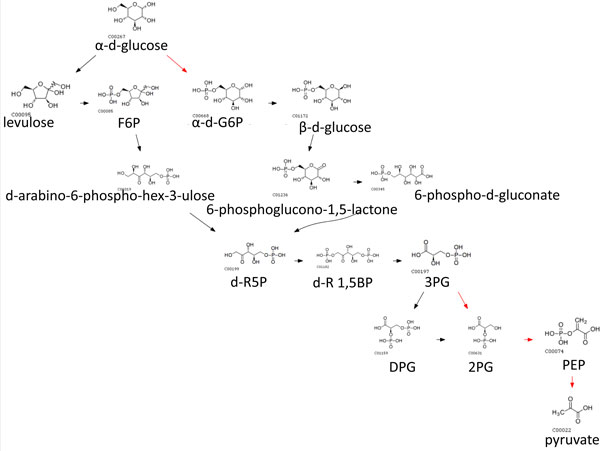
**Top 10 routes from glyucose to pyruvate obtained using MetaRoute**. (Native path not recovered).

**Figure 8:**
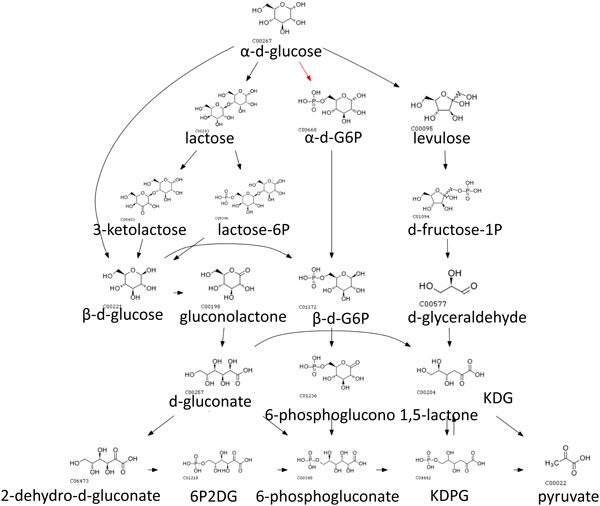
**Top 10 routes from glucose to pyruvate obtained using NEAT**. (Native path not recovered). Paths predicted using MRSD, Metaroute and NEAT do not recover the native route. Alternative paths also do not align well with the native path.

We also used weights and penalty scores in earler sections so that switching penalties reflected how different the switched species/pathways are. We set *w*_1 _= 0.33, *w*_2 _= 0.33 and *w*_3 _= 0.33, so that *γ*_1_, *γ*_2 _and *δ*_1 _contributes equally to an edge weight. The routes obtained from assigning dynamic penalties in CMPF aligned better to native pathways and alternative routes are only a slight deviation from native pathways, suggesting that such routes are more likely to happen in real biological systems. In contrast there is little alignment observed for other methods. For example, the glucose to pyruvate route with native path in red is shown in Figure 4, 5, 6, 7, 8.

**Figure 9:**
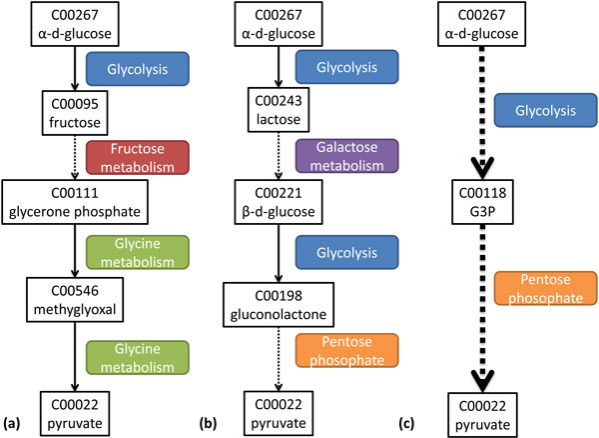
**(a) Path extracted from MRSD, (b) Path extracted from NEAT, (c) Path extracted from *CMPF_dynamic_***. The dotted edge represents additional reactions not shown in the same pathway. The width of the edge represents the number of reactions within the same pathway. A pathway trace is a sequence of pathways activated in a linear metabolic path represented by colored boxes.

A linear path can be also represented by tracing which pathways are used at each reaction step. This pathway trace intuitively tells us the transitions between pathways in a linear path (See Figure 9). Native paths have a short pathway trace because they often do not cross pathways since reactions in the same pathway perform a biologically efficient function. Our results show that paths produced by other methods not only deviate from the native path, they also have longer pathway traces. We chose glucose and pyruvate as source and target respectively because it is a well-studied metabolic process breaking down the carbon backbone in the glycolysis pathway.

There are two consequences to long pathway traces. The first is that pathway transitions 'hop' from one pathway to another. The number of consecutive reactions in the same pathway is small. We believe this that such transitions activates many different biological functions without achieving any specific purpose. For example, one of NEAT's predicted paths is shown in Figure 9b. The dotted lines represent additional reaction steps in the same pathway not shown in the figure. The width of the dotted edge represents the number of consecutive reaction steps in the same pathway. It starts by breaking down glucose (glycolysis pathway) and transits to converting and breaking down of lactose, half way through the process, the intermediate metabolite is converted back to a glucose analog, switching back to glycolysis followed by a transition to the pentose phosphate pathway. In contrast, CMPF prefers to stick to the same pathway until a switch is permissible, as indicated by the thicker dotted lines (See Figure 9c).

The second consequence is that a transition might be made to a non-relevant pathway. For example, one of MRSD's predicted paths is shown in Figure 9a. The reaction starts from breaking down glucose to producing fructose followed by a diversion to the glycine protein pathway before finally producing pyruvate. We think this is biologically not meaningful because breaking down of glucose into pyruvate is a simple function that does not involve anabolism or catabolism of amino-acids. While it is technically possible to obtain pyruvate from glucose by going through the protein pathway, it might make more sense to produce amino acids after transiting to the protein pathway rather than coming back to break down glucose. Instead, whenever a switch is permissible, CMPF prefers to switch to the most similar pathway based on our dynamic scoring function.

**Figure 10:**
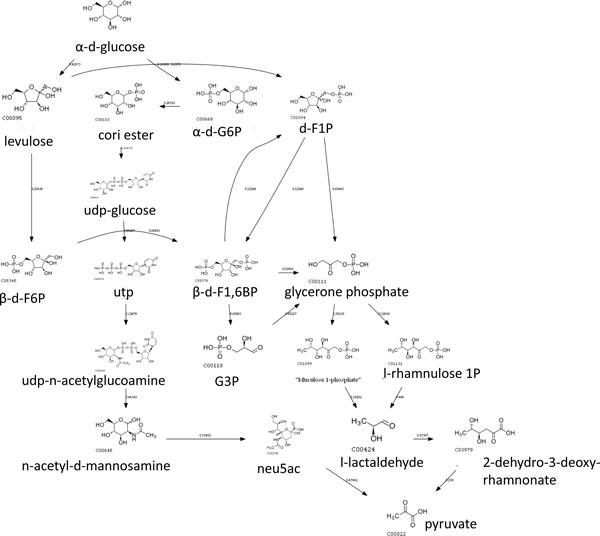
**Non native routes obtained using CMPF by avoiding the glycolysis pathway**.

Our method is also flexible to find non-native paths (if required) while incurring minimal pathway switches at the same time. For example, one can simply remove glycolysis from the set *P ϕ*(*r*1) so that future reactions will avoid switching to the glycolysis pathway. The switching minimized path from glucose to pyruvate without using the glycolysis pathway is shown in Figure 10. One of the paths can be achieved within the nucleotide sugar metabolism pathway. While paths that do not align well to native pathways might be spurious, alternative paths do give biological insights to the processes within the cell. Amongst these paths, those that have lesser switching might be more likely to be interesting as they circumvent the two biological consequences discussed above.

### Implementation

We build a compound-RPAIR bipartite 'super' graph representing the all reactions in the metabolic network from all species and pathways as described previously. The RPAIR database from KEGG is categorized into 'main', 'trans', 'cofac', 'ligase' and 'leave' depending on their roles in a chemical reaction [7]. To avoid currency compounds, we used main RPAIRs to construct the 'super' graph. This allows us to enumerate paths in a reasonable time since many irrelevant edges are excluded from the 'super' graph. However, we note that some 'trans' RPAIRs are present in native paths, suggesting that some 'trans' RPAIRs are important for pathfinding.

On the other hand, permitting edges from 'trans' RPAIRs make the exhaustive search significantly slower. To allow a more comprehensive search to run within reasonable time, we permitted 'trans' RPAIRs edges if they do not increase the graph branching factor by too much. To do this, we measure the increase in node connectivity after adding 'trans' RPAIR edges. The 'trans' RPAIR edges would be added only if they lie within one standard deviation from the median. This is a heuristic approach and thus may miss informative paths that include 'trans' RPAIRs.

We developed a software package to exhaustively traverse the graph up to a user specified depth threshold [see Additional file 1]. The user specifies the starting and final product as well as the weights for scoring paths. The paths are displayed using a well known graph visualization tool, GraphViz [13]. The software also allows users to highlight paths by their score ranking.

## Conclusion

The problem of predicting source target routes in a biological pathway depends on the users' searching criteria. We have shown in this paper that our proposed path scoring scheme gives users the alternative to find paths that minimizes class crossing and also allows users to evaluate predicted paths. Our scoring scheme is also sufficiently flexible to allow us to find routes with minimal switching between species or any other class that a reaction can participate in. We evaluate our method against other graph-based methods and demonstrate that paths ranked by our scoring scheme align better to native paths. This suggests that alternative paths predicted by our method might be more likely to occur in real biological systems.

## Availability and Requirements

**Project name: **CMPF

**Project homepage: **http://compbio.ddns.comp.nus.edu.sg:8080/cmpf/

**Operating System(s): **Platform independent. Windows system is tested

**Programming language: **Java

**Other requirements: **Java runtime environment 1.6 and above, at least 1GB of free RAM.

**License: **No

## Competing interests

The authors declare that they have no competing interests.

## Authors' contributions

KL derived and implemented the algorithms, performed the experiments, and drafted the manuscript. LW conceived, directed the project and revised the manuscript. All authors read and approved the final manuscript.

**Figure 11:**
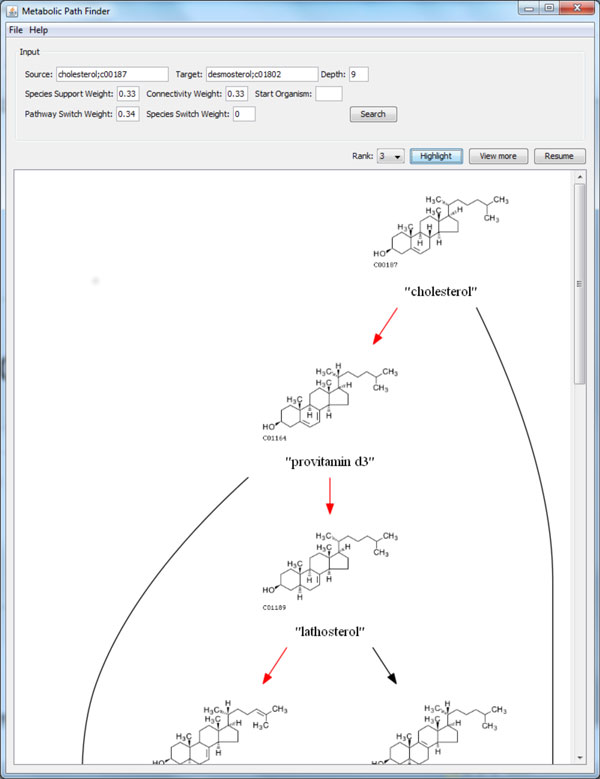
**A screenshot of CMPF**. Our software package includes a visualization tool. Paths are drawn with their molecular structure shown to allow users to visualize the change in chemical structure.

## Supplementary Material

Additional file 1 **Software package for CMPF**. Click here for file

## References

[B1] Blum T Kohlbacher O MetaRoute: fast search for relevant metabolic routes for interactive network navigation and visualization Bioinformatics 2008 24 18 2108 2109 http://bioinformatics.oxfordjournals.org/content/24/18/2108.abstract 10.1093/bioinformatics/btn360 18635573 PMC2530881

[B2] Croes D Couche F Wodak SJ van Helden J Metabolic PathFinding: inferring relevant pathways in biochemical networks 2005 33 suppl 2 W326 W330 10.1093/nar/gki437 PMC1160198 15980483

[B3] Rahman SA Advani P Schunk R Schrader R Schomburg D Metabolic pathway analysis web service (Pathway Hunter Tool at CUBIC) Bioinformatics 2005 21 7 1189 1193 http://bioinformatics.oxfordjournals.org/content/21/7/1189.abstract 10.1093/bioinformatics/bti116 15572476

[B4] Handorf T Ebenhöh O Heinrich R Expanding Metabolic Networks: Scopes of Compounds, Robustness, and Evolution Journal of Molecular Evolution 2005 61 498 512 http://dx.doi.org/10.1007/s00239-005-0027-1 [10.1007/s00239-005-0027-1] 10.1007/s00239-005-0027-1 16155745

[B5] Blum T Kohlbacher O Using atom mapping rules for an improved detection of relevant routes in weighted metabolic networks J Comput Biol 2008 15 6 565 576 http://dx.doi.org/10.1089/cmb.2008.0044 10.1089/cmb.2008.0044 18631021

[B6] Eppstein D Finding the *k *shortest paths Proc 35th Symp Foundations of Computer Science, IEEE 1994 154 165

[B7] Faust K Croes D van Helden J Metabolic Pathfinding Using RPAIR Annotation Journal of Molecular Biology 2009 388 2 390 414 http://dx.doi.org/10.1016/j.jmb.2009.03.006 10.1016/j.jmb.2009.03.006 19281817

[B8] Oh M Yamada T Hattori M Goto S Kanehisa M Systematic Analysis of Enzyme-Catalyzed Reaction Patterns and Prediction of Microbial Biodegradation Pathways Journal of Chemical Information and Modeling 2007 47 4 1702 1712 http://pubs.acs.org/doi/abs/10.1021/ci700006f 10.1021/ci700006f 17516640

[B9] Hattori M Okuno Y Goto S Kanehisa M Development of a Chemical Structure Comparison Method for Integrated Analysis of Chemical and Genomic Information in the Metabolic Pathways Journal of the American Chemical Society 2003 125 39 11853 11865 http://pubs.acs.org/doi/abs/10.1021/ja036030u 10.1021/ja036030u 14505407

[B10] Moriya Y Shigemizu D Hattori M Tokimatsu T Kotera M Goto S Kanehisa M PathPred: an enzyme-catalyzed metabolic pathway prediction server Nucleic Acids Research 2010 38 suppl 2 W138 W143 http://nar.oxfordjournals.org/content/38/suppl_2/W138.abstract 20435670 10.1093/nar/gkq318 PMC2896155

[B11] Xia D Zheng H Liu Z Li G Li J Hong J Zhao K MRSD: a web server for Metabolic Route Search and Design Bioinformatics 2011 27 11 1581 1582 http://bioinformatics.oxfordjournals.org/content/27/11/1581 10.1093/bioinformatics/btr160 21450713

[B12] Pey J Prada J Beasley J Planes F Path finding methods accounting for stoichiometry in metabolic networks Genome Biology 2011 12 5 R49 http://genomebiology.com/2011/12/5/R49 10.1186/gb-2011-12-5-r49 21619601 PMC3219972

[B13] Ellson J Gansner E Koutsofios L North S Woodhull G Description S Technologies L Graphviz open source graph drawing tools Lecture Notes in Computer Science, Springer-Verlag 2001 483 484

