# Supplementary figures and images for: CMPF: Class-switching minimized pathfinding in metabolic networks (part 2 of 2)
Source: BMC Bioinformatics. 2012 Dec 7;13(Suppl 17):S17. doi: 10.1186/1471-2105-13-S17-S17 (PMC3521384; doi:10.1186/1471-2105-13-S17-S17)

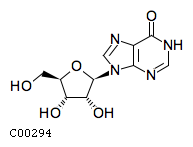

Supplement: Additional file 1 — Software package for CMPF. [file 1471-2105-13-S17-S17-S1.ZIP › img/C00294.gif]

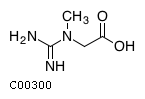

Supplement: Additional file 1 — Software package for CMPF. [file 1471-2105-13-S17-S17-S1.ZIP › img/C00300.gif]

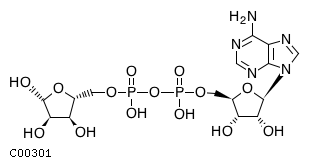

Supplement: Additional file 1 — Software package for CMPF. [file 1471-2105-13-S17-S17-S1.ZIP › img/C00301.gif]

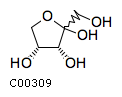

Supplement: Additional file 1 — Software package for CMPF. [file 1471-2105-13-S17-S17-S1.ZIP › img/C00309.gif]

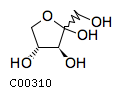

Supplement: Additional file 1 — Software package for CMPF. [file 1471-2105-13-S17-S17-S1.ZIP › img/C00310.gif]

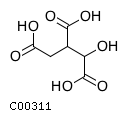

Supplement: Additional file 1 — Software package for CMPF. [file 1471-2105-13-S17-S17-S1.ZIP › img/C00311.gif]

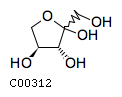

Supplement: Additional file 1 — Software package for CMPF. [file 1471-2105-13-S17-S17-S1.ZIP › img/C00312.gif]

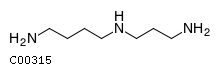

Supplement: Additional file 1 — Software package for CMPF. [file 1471-2105-13-S17-S17-S1.ZIP › img/C00315.gif]

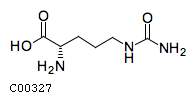

Supplement: Additional file 1 — Software package for CMPF. [file 1471-2105-13-S17-S17-S1.ZIP › img/C00327.gif]

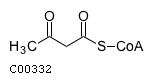

Supplement: Additional file 1 — Software package for CMPF. [file 1471-2105-13-S17-S17-S1.ZIP › img/C00332.gif]

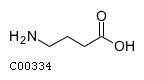

Supplement: Additional file 1 — Software package for CMPF. [file 1471-2105-13-S17-S17-S1.ZIP › img/C00334.gif]

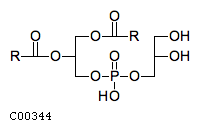

Supplement: Additional file 1 — Software package for CMPF. [file 1471-2105-13-S17-S17-S1.ZIP › img/C00344.gif]

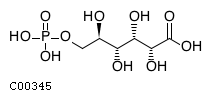

Supplement: Additional file 1 — Software package for CMPF. [file 1471-2105-13-S17-S17-S1.ZIP › img/C00345.gif]

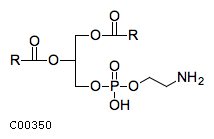

Supplement: Additional file 1 — Software package for CMPF. [file 1471-2105-13-S17-S17-S1.ZIP › img/C00350.gif]

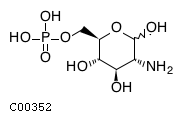

Supplement: Additional file 1 — Software package for CMPF. [file 1471-2105-13-S17-S17-S1.ZIP › img/C00352.gif]

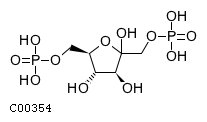

Supplement: Additional file 1 — Software package for CMPF. [file 1471-2105-13-S17-S17-S1.ZIP › img/C00354.gif]

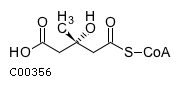

Supplement: Additional file 1 — Software package for CMPF. [file 1471-2105-13-S17-S17-S1.ZIP › img/C00356.gif]

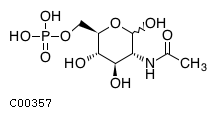

Supplement: Additional file 1 — Software package for CMPF. [file 1471-2105-13-S17-S17-S1.ZIP › img/C00357.gif]

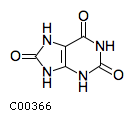

Supplement: Additional file 1 — Software package for CMPF. [file 1471-2105-13-S17-S17-S1.ZIP › img/C00366.gif]

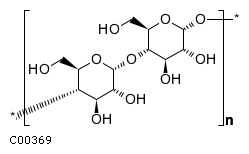

Supplement: Additional file 1 — Software package for CMPF. [file 1471-2105-13-S17-S17-S1.ZIP › img/C00369.gif]

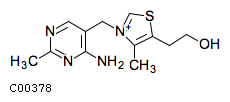

Supplement: Additional file 1 — Software package for CMPF. [file 1471-2105-13-S17-S17-S1.ZIP › img/C00378.gif]

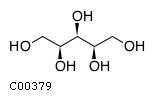

Supplement: Additional file 1 — Software package for CMPF. [file 1471-2105-13-S17-S17-S1.ZIP › img/C00379.gif]

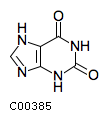

Supplement: Additional file 1 — Software package for CMPF. [file 1471-2105-13-S17-S17-S1.ZIP › img/C00385.gif]

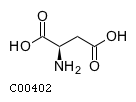

Supplement: Additional file 1 — Software package for CMPF. [file 1471-2105-13-S17-S17-S1.ZIP › img/C00402.gif]

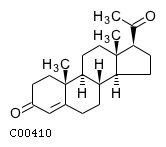

Supplement: Additional file 1 — Software package for CMPF. [file 1471-2105-13-S17-S17-S1.ZIP › img/C00410.gif]

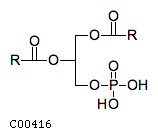

Supplement: Additional file 1 — Software package for CMPF. [file 1471-2105-13-S17-S17-S1.ZIP › img/C00416.gif]

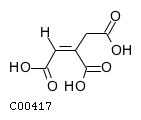

Supplement: Additional file 1 — Software package for CMPF. [file 1471-2105-13-S17-S17-S1.ZIP › img/C00417.gif]

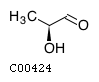

Supplement: Additional file 1 — Software package for CMPF. [file 1471-2105-13-S17-S17-S1.ZIP › img/C00424.gif]

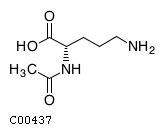

Supplement: Additional file 1 — Software package for CMPF. [file 1471-2105-13-S17-S17-S1.ZIP › img/C00437.gif]

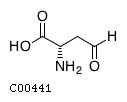

Supplement: Additional file 1 — Software package for CMPF. [file 1471-2105-13-S17-S17-S1.ZIP › img/C00441.gif]

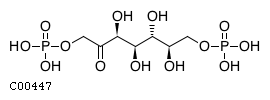

Supplement: Additional file 1 — Software package for CMPF. [file 1471-2105-13-S17-S17-S1.ZIP › img/C00447.gif]

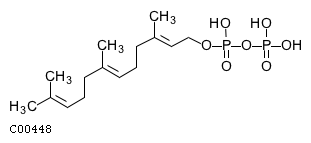

Supplement: Additional file 1 — Software package for CMPF. [file 1471-2105-13-S17-S17-S1.ZIP › img/C00448.gif]

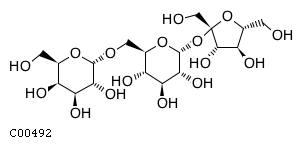

Supplement: Additional file 1 — Software package for CMPF. [file 1471-2105-13-S17-S17-S1.ZIP › img/C00492.gif]

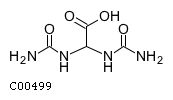

Supplement: Additional file 1 — Software package for CMPF. [file 1471-2105-13-S17-S17-S1.ZIP › img/C00499.gif]

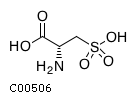

Supplement: Additional file 1 — Software package for CMPF. [file 1471-2105-13-S17-S17-S1.ZIP › img/C00506.gif]

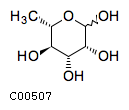

Supplement: Additional file 1 — Software package for CMPF. [file 1471-2105-13-S17-S17-S1.ZIP › img/C00507.gif]

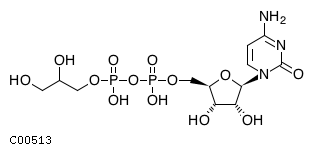

Supplement: Additional file 1 — Software package for CMPF. [file 1471-2105-13-S17-S17-S1.ZIP › img/C00513.gif]

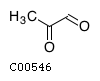

Supplement: Additional file 1 — Software package for CMPF. [file 1471-2105-13-S17-S17-S1.ZIP › img/C00546.gif]

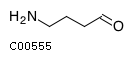

Supplement: Additional file 1 — Software package for CMPF. [file 1471-2105-13-S17-S17-S1.ZIP › img/C00555.gif]

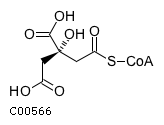

Supplement: Additional file 1 — Software package for CMPF. [file 1471-2105-13-S17-S17-S1.ZIP › img/C00566.gif]

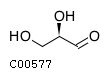

Supplement: Additional file 1 — Software package for CMPF. [file 1471-2105-13-S17-S17-S1.ZIP › img/C00577.gif]

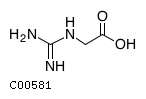

Supplement: Additional file 1 — Software package for CMPF. [file 1471-2105-13-S17-S17-S1.ZIP › img/C00581.gif]

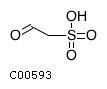

Supplement: Additional file 1 — Software package for CMPF. [file 1471-2105-13-S17-S17-S1.ZIP › img/C00593.gif]

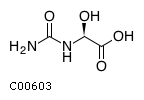

Supplement: Additional file 1 — Software package for CMPF. [file 1471-2105-13-S17-S17-S1.ZIP › img/C00603.gif]

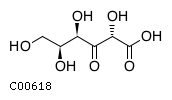

Supplement: Additional file 1 — Software package for CMPF. [file 1471-2105-13-S17-S17-S1.ZIP › img/C00618.gif]

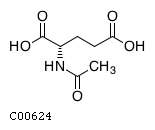

Supplement: Additional file 1 — Software package for CMPF. [file 1471-2105-13-S17-S17-S1.ZIP › img/C00624.gif]

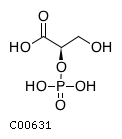

Supplement: Additional file 1 — Software package for CMPF. [file 1471-2105-13-S17-S17-S1.ZIP › img/C00631.gif]

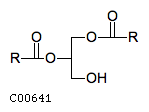

Supplement: Additional file 1 — Software package for CMPF. [file 1471-2105-13-S17-S17-S1.ZIP › img/C00641.gif]

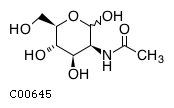

Supplement: Additional file 1 — Software package for CMPF. [file 1471-2105-13-S17-S17-S1.ZIP › img/C00645.gif]

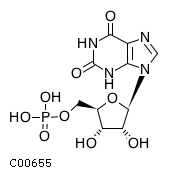

Supplement: Additional file 1 — Software package for CMPF. [file 1471-2105-13-S17-S17-S1.ZIP › img/C00655.gif]

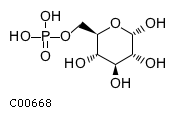

Supplement: Additional file 1 — Software package for CMPF. [file 1471-2105-13-S17-S17-S1.ZIP › img/C00668.gif]

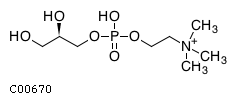

Supplement: Additional file 1 — Software package for CMPF. [file 1471-2105-13-S17-S17-S1.ZIP › img/C00670.gif]

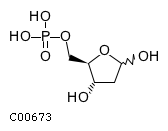

Supplement: Additional file 1 — Software package for CMPF. [file 1471-2105-13-S17-S17-S1.ZIP › img/C00673.gif]

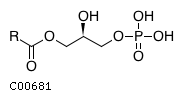

Supplement: Additional file 1 — Software package for CMPF. [file 1471-2105-13-S17-S17-S1.ZIP › img/C00681.gif]

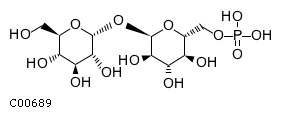

Supplement: Additional file 1 — Software package for CMPF. [file 1471-2105-13-S17-S17-S1.ZIP › img/C00689.gif]

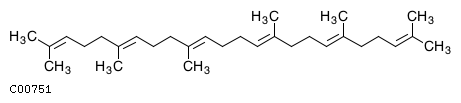

Supplement: Additional file 1 — Software package for CMPF. [file 1471-2105-13-S17-S17-S1.ZIP › img/C00751.gif]

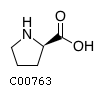

Supplement: Additional file 1 — Software package for CMPF. [file 1471-2105-13-S17-S17-S1.ZIP › img/C00763.gif]

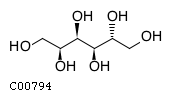

Supplement: Additional file 1 — Software package for CMPF. [file 1471-2105-13-S17-S17-S1.ZIP › img/C00794.gif]

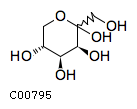

Supplement: Additional file 1 — Software package for CMPF. [file 1471-2105-13-S17-S17-S1.ZIP › img/C00795.gif]

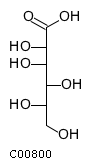

Supplement: Additional file 1 — Software package for CMPF. [file 1471-2105-13-S17-S17-S1.ZIP › img/C00800.gif]

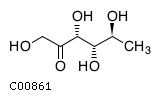

Supplement: Additional file 1 — Software package for CMPF. [file 1471-2105-13-S17-S17-S1.ZIP › img/C00861.gif]

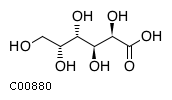

Supplement: Additional file 1 — Software package for CMPF. [file 1471-2105-13-S17-S17-S1.ZIP › img/C00880.gif]

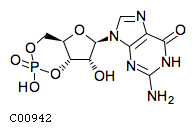

Supplement: Additional file 1 — Software package for CMPF. [file 1471-2105-13-S17-S17-S1.ZIP › img/C00942.gif]

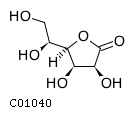

Supplement: Additional file 1 — Software package for CMPF. [file 1471-2105-13-S17-S17-S1.ZIP › img/C01040.gif]

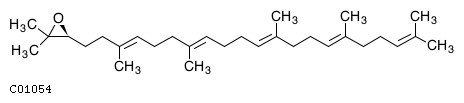

Supplement: Additional file 1 — Software package for CMPF. [file 1471-2105-13-S17-S17-S1.ZIP › img/C01054.gif]

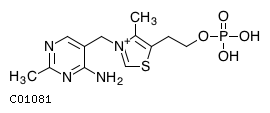

Supplement: Additional file 1 — Software package for CMPF. [file 1471-2105-13-S17-S17-S1.ZIP › img/C01081.gif]

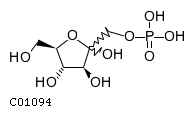

Supplement: Additional file 1 — Software package for CMPF. [file 1471-2105-13-S17-S17-S1.ZIP › img/C01094.gif]

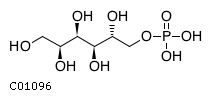

Supplement: Additional file 1 — Software package for CMPF. [file 1471-2105-13-S17-S17-S1.ZIP › img/C01096.gif]

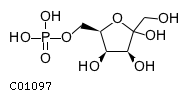

Supplement: Additional file 1 — Software package for CMPF. [file 1471-2105-13-S17-S17-S1.ZIP › img/C01097.gif]

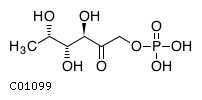

Supplement: Additional file 1 — Software package for CMPF. [file 1471-2105-13-S17-S17-S1.ZIP › img/C01099.gif]

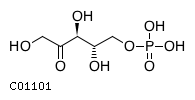

Supplement: Additional file 1 — Software package for CMPF. [file 1471-2105-13-S17-S17-S1.ZIP › img/C01101.gif]

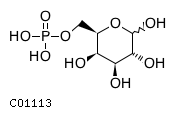

Supplement: Additional file 1 — Software package for CMPF. [file 1471-2105-13-S17-S17-S1.ZIP › img/C01113.gif]

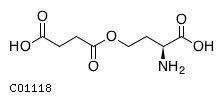

Supplement: Additional file 1 — Software package for CMPF. [file 1471-2105-13-S17-S17-S1.ZIP › img/C01118.gif]

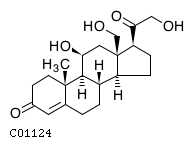

Supplement: Additional file 1 — Software package for CMPF. [file 1471-2105-13-S17-S17-S1.ZIP › img/C01124.gif]

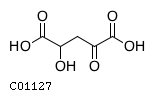

Supplement: Additional file 1 — Software package for CMPF. [file 1471-2105-13-S17-S17-S1.ZIP › img/C01127.gif]

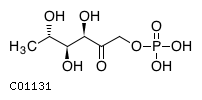

Supplement: Additional file 1 — Software package for CMPF. [file 1471-2105-13-S17-S17-S1.ZIP › img/C01131.gif]

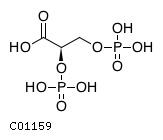

Supplement: Additional file 1 — Software package for CMPF. [file 1471-2105-13-S17-S17-S1.ZIP › img/C01159.gif]

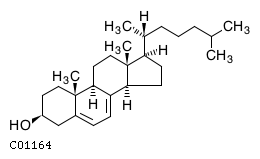

Supplement: Additional file 1 — Software package for CMPF. [file 1471-2105-13-S17-S17-S1.ZIP › img/C01164.gif]

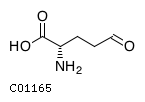

Supplement: Additional file 1 — Software package for CMPF. [file 1471-2105-13-S17-S17-S1.ZIP › img/C01165.gif]

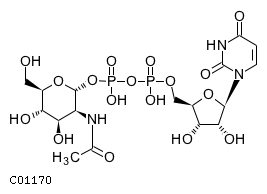

Supplement: Additional file 1 — Software package for CMPF. [file 1471-2105-13-S17-S17-S1.ZIP › img/C01170.gif]

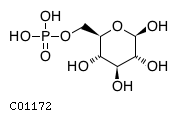

Supplement: Additional file 1 — Software package for CMPF. [file 1471-2105-13-S17-S17-S1.ZIP › img/C01172.gif]
