# Supplementary figures and images for: CMPF: Class-switching minimized pathfinding in metabolic networks (part 1 of 2)
Source: BMC Bioinformatics. 2012 Dec 7;13(Suppl 17):S17. doi: 10.1186/1471-2105-13-S17-S17 (PMC3521384; doi:10.1186/1471-2105-13-S17-S17)

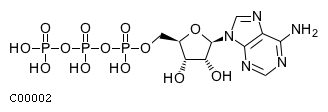

Supplement: Additional file 1 — Software package for CMPF. [file 1471-2105-13-S17-S17-S1.ZIP › img/C00002.gif]

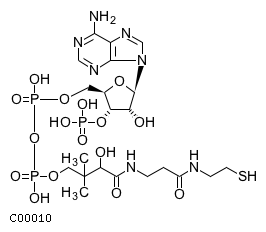

Supplement: Additional file 1 — Software package for CMPF. [file 1471-2105-13-S17-S17-S1.ZIP › img/C00010.gif]

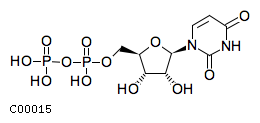

Supplement: Additional file 1 — Software package for CMPF. [file 1471-2105-13-S17-S17-S1.ZIP › img/C00015.gif]

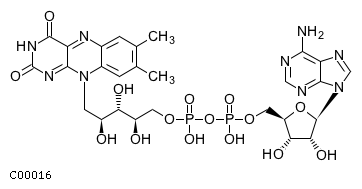

Supplement: Additional file 1 — Software package for CMPF. [file 1471-2105-13-S17-S17-S1.ZIP › img/C00016.gif]

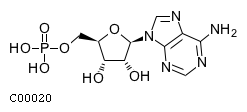

Supplement: Additional file 1 — Software package for CMPF. [file 1471-2105-13-S17-S17-S1.ZIP › img/C00020.gif]

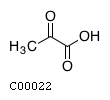

Supplement: Additional file 1 — Software package for CMPF. [file 1471-2105-13-S17-S17-S1.ZIP › img/C00022.gif]

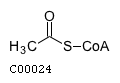

Supplement: Additional file 1 — Software package for CMPF. [file 1471-2105-13-S17-S17-S1.ZIP › img/C00024.gif]

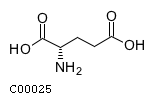

Supplement: Additional file 1 — Software package for CMPF. [file 1471-2105-13-S17-S17-S1.ZIP › img/C00025.gif]

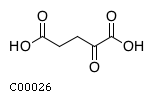

Supplement: Additional file 1 — Software package for CMPF. [file 1471-2105-13-S17-S17-S1.ZIP › img/C00026.gif]

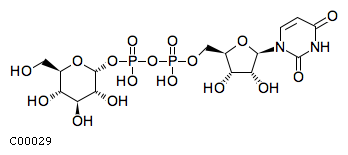

Supplement: Additional file 1 — Software package for CMPF. [file 1471-2105-13-S17-S17-S1.ZIP › img/C00029.gif]

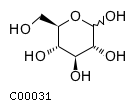

Supplement: Additional file 1 — Software package for CMPF. [file 1471-2105-13-S17-S17-S1.ZIP › img/C00031.gif]

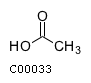

Supplement: Additional file 1 — Software package for CMPF. [file 1471-2105-13-S17-S17-S1.ZIP › img/C00033.gif]

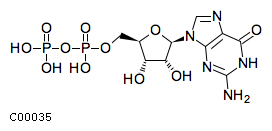

Supplement: Additional file 1 — Software package for CMPF. [file 1471-2105-13-S17-S17-S1.ZIP › img/C00035.gif]

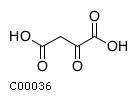

Supplement: Additional file 1 — Software package for CMPF. [file 1471-2105-13-S17-S17-S1.ZIP › img/C00036.gif]

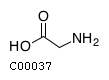

Supplement: Additional file 1 — Software package for CMPF. [file 1471-2105-13-S17-S17-S1.ZIP › img/C00037.gif]

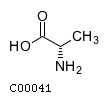

Supplement: Additional file 1 — Software package for CMPF. [file 1471-2105-13-S17-S17-S1.ZIP › img/C00041.gif]

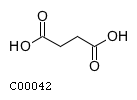

Supplement: Additional file 1 — Software package for CMPF. [file 1471-2105-13-S17-S17-S1.ZIP › img/C00042.gif]

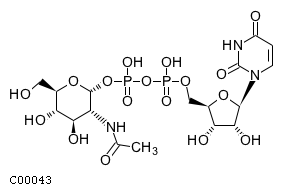

Supplement: Additional file 1 — Software package for CMPF. [file 1471-2105-13-S17-S17-S1.ZIP › img/C00043.gif]

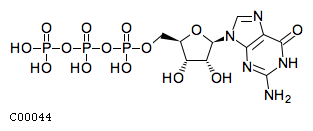

Supplement: Additional file 1 — Software package for CMPF. [file 1471-2105-13-S17-S17-S1.ZIP › img/C00044.gif]

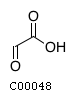

Supplement: Additional file 1 — Software package for CMPF. [file 1471-2105-13-S17-S17-S1.ZIP › img/C00048.gif]

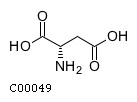

Supplement: Additional file 1 — Software package for CMPF. [file 1471-2105-13-S17-S17-S1.ZIP › img/C00049.gif]

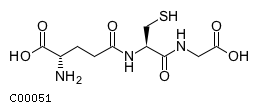

Supplement: Additional file 1 — Software package for CMPF. [file 1471-2105-13-S17-S17-S1.ZIP › img/C00051.gif]

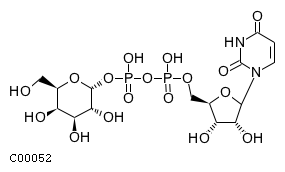

Supplement: Additional file 1 — Software package for CMPF. [file 1471-2105-13-S17-S17-S1.ZIP › img/C00052.gif]

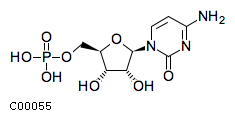

Supplement: Additional file 1 — Software package for CMPF. [file 1471-2105-13-S17-S17-S1.ZIP › img/C00055.gif]

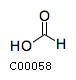

Supplement: Additional file 1 — Software package for CMPF. [file 1471-2105-13-S17-S17-S1.ZIP › img/C00058.gif]

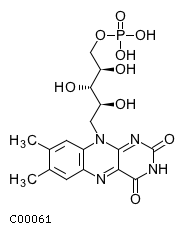

Supplement: Additional file 1 — Software package for CMPF. [file 1471-2105-13-S17-S17-S1.ZIP › img/C00061.gif]

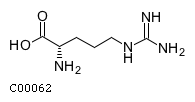

Supplement: Additional file 1 — Software package for CMPF. [file 1471-2105-13-S17-S17-S1.ZIP › img/C00062.gif]

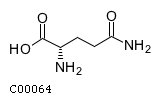

Supplement: Additional file 1 — Software package for CMPF. [file 1471-2105-13-S17-S17-S1.ZIP › img/C00064.gif]

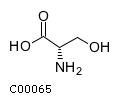

Supplement: Additional file 1 — Software package for CMPF. [file 1471-2105-13-S17-S17-S1.ZIP › img/C00065.gif]

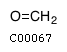

Supplement: Additional file 1 — Software package for CMPF. [file 1471-2105-13-S17-S17-S1.ZIP › img/C00067.gif]

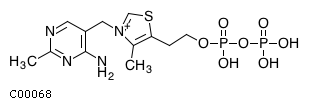

Supplement: Additional file 1 — Software package for CMPF. [file 1471-2105-13-S17-S17-S1.ZIP › img/C00068.gif]

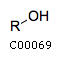

Supplement: Additional file 1 — Software package for CMPF. [file 1471-2105-13-S17-S17-S1.ZIP › img/C00069.gif]

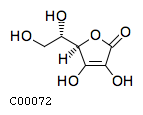

Supplement: Additional file 1 — Software package for CMPF. [file 1471-2105-13-S17-S17-S1.ZIP › img/C00072.gif]

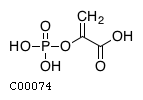

Supplement: Additional file 1 — Software package for CMPF. [file 1471-2105-13-S17-S17-S1.ZIP › img/C00074.gif]

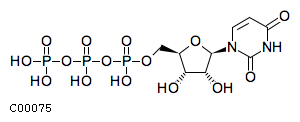

Supplement: Additional file 1 — Software package for CMPF. [file 1471-2105-13-S17-S17-S1.ZIP › img/C00075.gif]

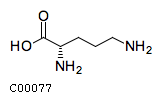

Supplement: Additional file 1 — Software package for CMPF. [file 1471-2105-13-S17-S17-S1.ZIP › img/C00077.gif]

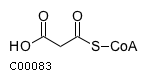

Supplement: Additional file 1 — Software package for CMPF. [file 1471-2105-13-S17-S17-S1.ZIP › img/C00083.gif]

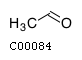

Supplement: Additional file 1 — Software package for CMPF. [file 1471-2105-13-S17-S17-S1.ZIP › img/C00084.gif]

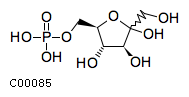

Supplement: Additional file 1 — Software package for CMPF. [file 1471-2105-13-S17-S17-S1.ZIP › img/C00085.gif]

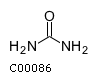

Supplement: Additional file 1 — Software package for CMPF. [file 1471-2105-13-S17-S17-S1.ZIP › img/C00086.gif]

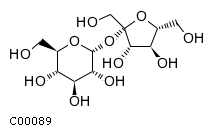

Supplement: Additional file 1 — Software package for CMPF. [file 1471-2105-13-S17-S17-S1.ZIP › img/C00089.gif]

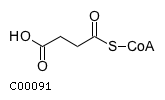

Supplement: Additional file 1 — Software package for CMPF. [file 1471-2105-13-S17-S17-S1.ZIP › img/C00091.gif]

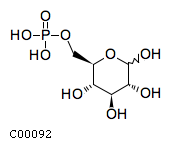

Supplement: Additional file 1 — Software package for CMPF. [file 1471-2105-13-S17-S17-S1.ZIP › img/C00092.gif]

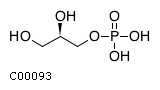

Supplement: Additional file 1 — Software package for CMPF. [file 1471-2105-13-S17-S17-S1.ZIP › img/C00093.gif]

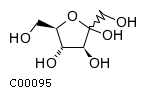

Supplement: Additional file 1 — Software package for CMPF. [file 1471-2105-13-S17-S17-S1.ZIP › img/C00095.gif]

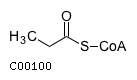

Supplement: Additional file 1 — Software package for CMPF. [file 1471-2105-13-S17-S17-S1.ZIP › img/C00100.gif]

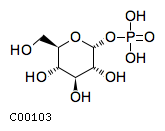

Supplement: Additional file 1 — Software package for CMPF. [file 1471-2105-13-S17-S17-S1.ZIP › img/C00103.gif]

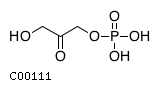

Supplement: Additional file 1 — Software package for CMPF. [file 1471-2105-13-S17-S17-S1.ZIP › img/C00111.gif]

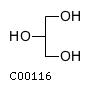

Supplement: Additional file 1 — Software package for CMPF. [file 1471-2105-13-S17-S17-S1.ZIP › img/C00116.gif]

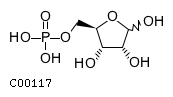

Supplement: Additional file 1 — Software package for CMPF. [file 1471-2105-13-S17-S17-S1.ZIP › img/C00117.gif]

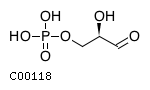

Supplement: Additional file 1 — Software package for CMPF. [file 1471-2105-13-S17-S17-S1.ZIP › img/C00118.gif]

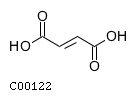

Supplement: Additional file 1 — Software package for CMPF. [file 1471-2105-13-S17-S17-S1.ZIP › img/C00122.gif]

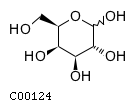

Supplement: Additional file 1 — Software package for CMPF. [file 1471-2105-13-S17-S17-S1.ZIP › img/C00124.gif]

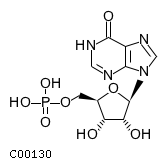

Supplement: Additional file 1 — Software package for CMPF. [file 1471-2105-13-S17-S17-S1.ZIP › img/C00130.gif]

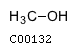

Supplement: Additional file 1 — Software package for CMPF. [file 1471-2105-13-S17-S17-S1.ZIP › img/C00132.gif]

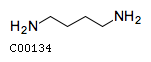

Supplement: Additional file 1 — Software package for CMPF. [file 1471-2105-13-S17-S17-S1.ZIP › img/C00134.gif]

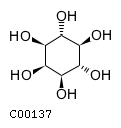

Supplement: Additional file 1 — Software package for CMPF. [file 1471-2105-13-S17-S17-S1.ZIP › img/C00137.gif]

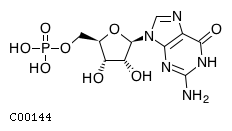

Supplement: Additional file 1 — Software package for CMPF. [file 1471-2105-13-S17-S17-S1.ZIP › img/C00144.gif]

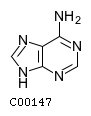

Supplement: Additional file 1 — Software package for CMPF. [file 1471-2105-13-S17-S17-S1.ZIP › img/C00147.gif]

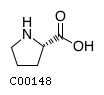

Supplement: Additional file 1 — Software package for CMPF. [file 1471-2105-13-S17-S17-S1.ZIP › img/C00148.gif]

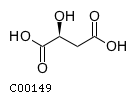

Supplement: Additional file 1 — Software package for CMPF. [file 1471-2105-13-S17-S17-S1.ZIP › img/C00149.gif]

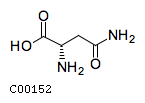

Supplement: Additional file 1 — Software package for CMPF. [file 1471-2105-13-S17-S17-S1.ZIP › img/C00152.gif]

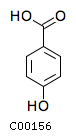

Supplement: Additional file 1 — Software package for CMPF. [file 1471-2105-13-S17-S17-S1.ZIP › img/C00156.gif]

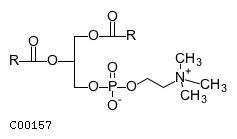

Supplement: Additional file 1 — Software package for CMPF. [file 1471-2105-13-S17-S17-S1.ZIP › img/C00157.gif]

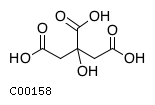

Supplement: Additional file 1 — Software package for CMPF. [file 1471-2105-13-S17-S17-S1.ZIP › img/C00158.gif]

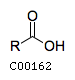

Supplement: Additional file 1 — Software package for CMPF. [file 1471-2105-13-S17-S17-S1.ZIP › img/C00162.gif]

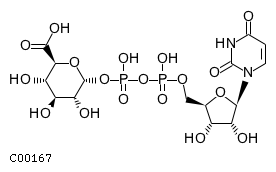

Supplement: Additional file 1 — Software package for CMPF. [file 1471-2105-13-S17-S17-S1.ZIP › img/C00167.gif]

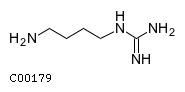

Supplement: Additional file 1 — Software package for CMPF. [file 1471-2105-13-S17-S17-S1.ZIP › img/C00179.gif]

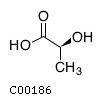

Supplement: Additional file 1 — Software package for CMPF. [file 1471-2105-13-S17-S17-S1.ZIP › img/C00186.gif]

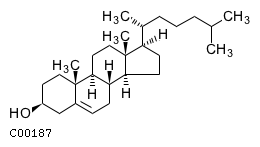

Supplement: Additional file 1 — Software package for CMPF. [file 1471-2105-13-S17-S17-S1.ZIP › img/C00187.gif]

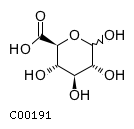

Supplement: Additional file 1 — Software package for CMPF. [file 1471-2105-13-S17-S17-S1.ZIP › img/C00191.gif]

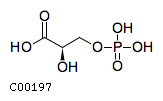

Supplement: Additional file 1 — Software package for CMPF. [file 1471-2105-13-S17-S17-S1.ZIP › img/C00197.gif]

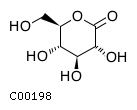

Supplement: Additional file 1 — Software package for CMPF. [file 1471-2105-13-S17-S17-S1.ZIP › img/C00198.gif]

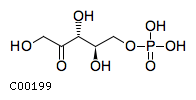

Supplement: Additional file 1 — Software package for CMPF. [file 1471-2105-13-S17-S17-S1.ZIP › img/C00199.gif]

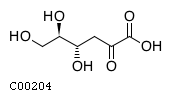

Supplement: Additional file 1 — Software package for CMPF. [file 1471-2105-13-S17-S17-S1.ZIP › img/C00204.gif]

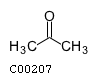

Supplement: Additional file 1 — Software package for CMPF. [file 1471-2105-13-S17-S17-S1.ZIP › img/C00207.gif]

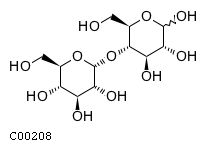

Supplement: Additional file 1 — Software package for CMPF. [file 1471-2105-13-S17-S17-S1.ZIP › img/C00208.gif]

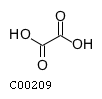

Supplement: Additional file 1 — Software package for CMPF. [file 1471-2105-13-S17-S17-S1.ZIP › img/C00209.gif]

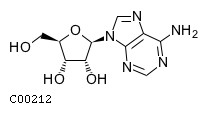

Supplement: Additional file 1 — Software package for CMPF. [file 1471-2105-13-S17-S17-S1.ZIP › img/C00212.gif]

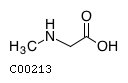

Supplement: Additional file 1 — Software package for CMPF. [file 1471-2105-13-S17-S17-S1.ZIP › img/C00213.gif]

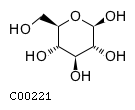

Supplement: Additional file 1 — Software package for CMPF. [file 1471-2105-13-S17-S17-S1.ZIP › img/C00221.gif]

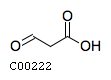

Supplement: Additional file 1 — Software package for CMPF. [file 1471-2105-13-S17-S17-S1.ZIP › img/C00222.gif]

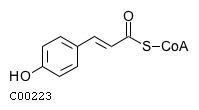

Supplement: Additional file 1 — Software package for CMPF. [file 1471-2105-13-S17-S17-S1.ZIP › img/C00223.gif]

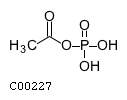

Supplement: Additional file 1 — Software package for CMPF. [file 1471-2105-13-S17-S17-S1.ZIP › img/C00227.gif]

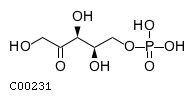

Supplement: Additional file 1 — Software package for CMPF. [file 1471-2105-13-S17-S17-S1.ZIP › img/C00231.gif]

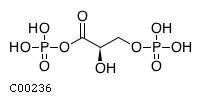

Supplement: Additional file 1 — Software package for CMPF. [file 1471-2105-13-S17-S17-S1.ZIP › img/C00236.gif]

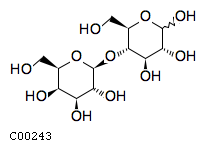

Supplement: Additional file 1 — Software package for CMPF. [file 1471-2105-13-S17-S17-S1.ZIP › img/C00243.gif]

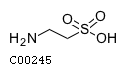

Supplement: Additional file 1 — Software package for CMPF. [file 1471-2105-13-S17-S17-S1.ZIP › img/C00245.gif]

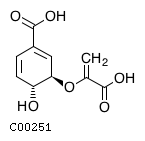

Supplement: Additional file 1 — Software package for CMPF. [file 1471-2105-13-S17-S17-S1.ZIP › img/C00251.gif]

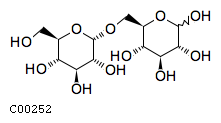

Supplement: Additional file 1 — Software package for CMPF. [file 1471-2105-13-S17-S17-S1.ZIP › img/C00252.gif]

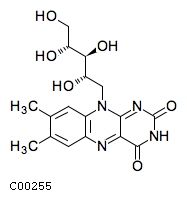

Supplement: Additional file 1 — Software package for CMPF. [file 1471-2105-13-S17-S17-S1.ZIP › img/C00255.gif]

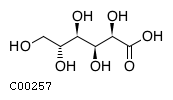

Supplement: Additional file 1 — Software package for CMPF. [file 1471-2105-13-S17-S17-S1.ZIP › img/C00257.gif]

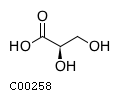

Supplement: Additional file 1 — Software package for CMPF. [file 1471-2105-13-S17-S17-S1.ZIP › img/C00258.gif]

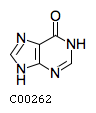

Supplement: Additional file 1 — Software package for CMPF. [file 1471-2105-13-S17-S17-S1.ZIP › img/C00262.gif]

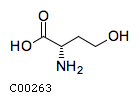

Supplement: Additional file 1 — Software package for CMPF. [file 1471-2105-13-S17-S17-S1.ZIP › img/C00263.gif]

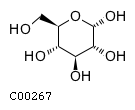

Supplement: Additional file 1 — Software package for CMPF. [file 1471-2105-13-S17-S17-S1.ZIP › img/C00267.gif]

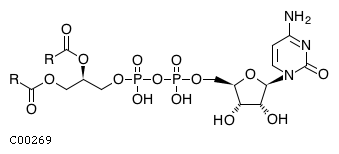

Supplement: Additional file 1 — Software package for CMPF. [file 1471-2105-13-S17-S17-S1.ZIP › img/C00269.gif]

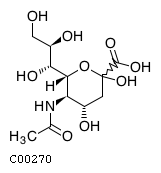

Supplement: Additional file 1 — Software package for CMPF. [file 1471-2105-13-S17-S17-S1.ZIP › img/C00270.gif]

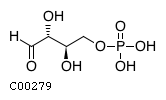

Supplement: Additional file 1 — Software package for CMPF. [file 1471-2105-13-S17-S17-S1.ZIP › img/C00279.gif]

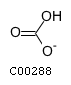

Supplement: Additional file 1 — Software package for CMPF. [file 1471-2105-13-S17-S17-S1.ZIP › img/C00288.gif]
